# Impact of SARS-CoV-2 infection on liver disease

**DOI:** 10.1515/almed-2022-0037

**Published:** 2022-06-02

**Authors:** Sergio Salgüero Fernández, Pablo Gabriel Medina, Alejandro Almería Lafuente, María Antonieta Ballesteros Vizoso, Angielys Zamora Trillo, Gregori Casals Mercadal, Gemma Solé Enrech, Marta Lalana Garcés, Armando R. Guerra Ruiz, Oihana Ortiz Pastor, Manuel Morales Ruiz

**Affiliations:** Biochemistry of Liver Disease Commision–Spanish Society of Laboratory Medicine (SEQC-ML), Alcorcon, Spain; Service of Clinical Analysis, Hospital Universitario Fundación Alcorcón, Madrid, Spain; Department of Clinical Biochemistry, Hospital Universitari Vall d’Hebron, Barcelona, Spain; Department of Clinical Biochemistry, Hospital Royo Villanova, Zaragoza, Spain; Service of Clinical Analysis, Hospital Universitario Son Espases, Palma de Mallorca, Spain; Department of Clinical Biochemistry, Hospital General Universitario Gregorio Marañón, Madrid, Spain; Service of Biochemistry and Molecular Genetics, CDB, Hospital Clínic de Barcelona, IDIBAPS, CIBEREHD, Barcelona, Spain; Laboratory Service UDIAT-CD, Corporació Sanitaria Parc Taulí, Sabadell, Spain; Service of Clinical Analysis, Hospital de Barbastro, Huesca, Spain; Service of Clinical Analysis, Hospital Universitario Marqués de Valdecilla, Santander, Spain; Department of Clinical Biochemistry, Hospital Universitario Miguel Servet, Zaragoza, Spain; Service of Biochemistry and Molecular Genetics, Department of Biomedicine of the Faculty of Medicine and Health Sciences, Hospital Clinic de Barcelona, IDIBAPS, CIBERehd, University of Barcelona, Barcelona, Spain

**Keywords:** liver injury, transaminase, virus

## Abstract

**Introduction:**

Abnormal liver biochemistry is not a rare finding in the context of SARS-CoV-2 infection, regardless of patients having pre-existing chronic disease or not

**Content:**

This review examines the current body of knowledge on the relationship between COVID-19 and liver injury, which is frequently found in this setting

**Summary:**

Although the pathogenesis of liver injury is not fully understood, it has been suggested to be the result of a combination of multiple factors. These include direct injury caused by the virus, immune system hyperactivation, ischemic and drug-induced injury. The prognostic valor of these alterations is also the subject of intense research. Due to their potential impact, these alterations require proper management and treatment, especially in patients with chronic liver disease or liver transplant recipients.

**Outlook:**

Some aspects associated with liver injury during COVID-19, especially in severe presentations, are not well understood. Studies assessing the clinical impact of COVID-19 on the healthy or diseased liver may help adjust treatment and immunization guidelines to the profile of the patient.

## Introduction

The disease caused by SARS-CoV-2 infection, named COVID-19, rapidly spread worldwide causing a pandemic with unprecedented clinical, humanitarian and economic effects in modern age [1, 2]. The number of updated cases and deaths can be monitored through a platform created by the John Hopkins University (https://coronavirus.jhu.edu/map.html). Data collected in the European Union reveal that 30% of cases required hospitalization, whereas 4% progressed to critical disease and needed ICU care [3]. Mortality is substantially high in patients with advanced age and pre-existing comorbidities, including obesity, cardiovascular diseases, and diabetes [4]. Data evolve as the pandemic progressed, with new variants emerging with a lower pathogenicity [5].

Although the cause of death generally is acute respiratory failure secondary to diffuse alveolar damage, the liver plays a key role during infection [6].

On the one hand, SARS-CoV-2 infection induces an elevation of markers of liver injury in patients without a previous history of liver dysfunction. These markers tend to rise as the duration of hospitalization and disease severity increase. In this context, there is cumulative evidence that levels of alanine aminotransferase (ALT), aspartate aminotransferase (AST), total bilirubin and gamma-glutamyl transferase (GGT) increase three times above the upper limit of normality (UPN) [7, 8]. The elevation of these markers of liver injury in COVID-19 patients is partially explained by the drugs used for the management of the disease, which supports liver biochemistry monitoring in the context of COVID-19.

On the other hand, the evidence collected in international multicentric registers (COVID-Hep.net and COVIDCirrhosis.org) demonstrates that chronic liver disease is another risk factor for the development of severe COVID-19 [9]. The evidence available suggests that patients with cirrhosis are at a higher risk for hepatic decompensation and COVID-19-related death, especially in the presence of advanced alcohol-related cirrhosis (9). SARS-CoV-2 tropism to cholangiocytes [10], cirrhosis-associated immune dysfunction [11], and other risk factors in these patients may explain the high mortality associated with complications of liver dysfunction.

In view of the evidence available demonstrating that COVID-19 has a negative impact on liver function, the leading scientific societies in the field of liver diseases (American Association for the Study of Liver Diseases o AASLD, European Association for the Study of the Liver o EASL and the Asian Pacific Association for the Study of the Liver or APASL) have issued their position papers where they provide guidelines for the management of liver injury secondary to COVID-19 infection [12], [13], [14].

In this line, this review addresses the pathogenesis of SARS-CoV-2 infection and its impact on liver dysfunction, and evaluate the follow-up needs of patients with pre-existing liver disease.

## Abnormal liver biochemistry

Abnormal liver biochemistry is frequent in SARS-CoV-2 infection, with occurrence ranging from 15 to 65% [15]. Indeed, there is evidence of liver injury in patients infected with another two highly pathogenic coronaviruses, SARS-CoV and MERS-CoV [16].

The most common finding is a slight elevation (i.e. 1–2 times the ULN) of cytolytic enzymes AST (38–63% of patients) and ALT (29–39%). Less frequently, some patients exhibit transaminase levels more than 5 times above the ULN, sometimes resulting in severe acute hepatitis [17].

AST is generally more elevated than ALT, which could indicate a non-hepatic lesion (i.e. myositis) [17]. However, AST levels are positively related to ALT levels, but not with those of rhabdomyolysis such as creatine kinase, or systemic inflammation, such as ferritin or C-reactive protein (CRP), which suggests the presence of liver injury [15]. Although the underlying mechanism for the preferential elevation of AST levels is unclear, mitochondrial dysfunction, steatosis and impaired liver perfusion may be involved [15].

In contrast, levels of alkaline phosphatase (6% of patients) and GGT (21%) are rarely elevated, and total bilirubin generally remains within normal limits or is only slightly elevated [15, 17].

The prognostic value of these biochemical alterations has not been fully elucidated. On the one hand, there is evidence that liver injury is more frequent in the context of severe COVID-19, as compared to mild disease [15, 17, 18]. Some authors establish a correlation between elevation of markers of liver function and a poor prognosis, defined as ICU admission, need for mechanical ventilation or mortality [15, 17, 18]. Finally, hypoalbuminemia has also been associated with poor prognosis as an unspecific marker of disease severity [15, 17].

This association, however, has not been confirmed in other studies, which reveal that, although liver injury is frequent, it may not have a significant impact on clinical outcomes and divert attention from the crucial goal of controlling generalized immune dysfunction [19, 20].

**Figure 1:**
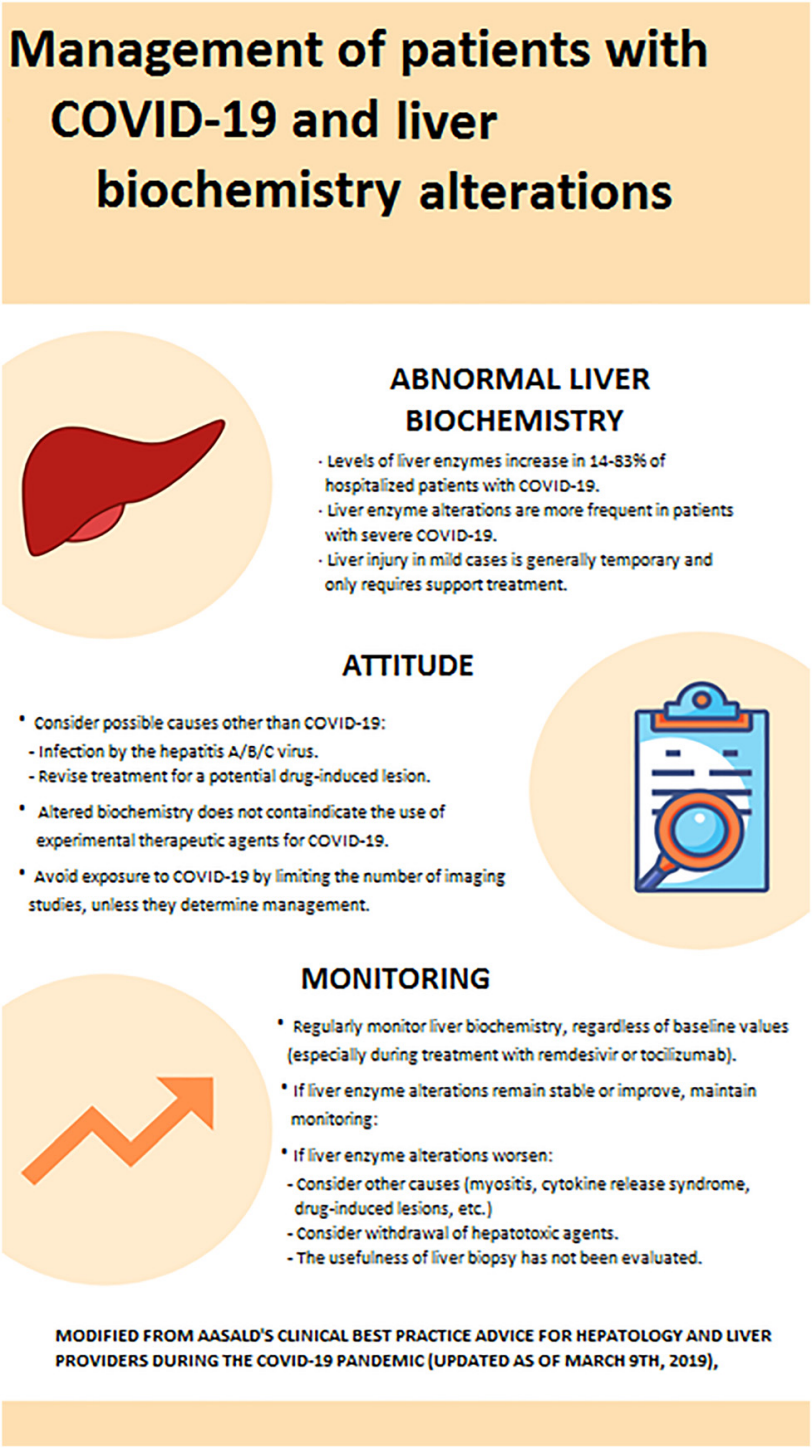
Guidelines for the management of patients with COVID-9 and description of liver biochemistry alterations (modified from [15]).

Liver injury in mild COVID-19 is generally transient and only requires support treatment, without a specific treatment being necessary. In addition, it is difficult to determine whether abnormal liver biochemistry is secondary either to viral infection and its complications (myositis, cytokine release syndrome, ischemia/hypotension) or to the treatment administered for COVID-19. Given the inconsistent evidence available about the relevance of abnormal liver biochemistry, the AASLD advocates in its consensus statement for a watchful attitude by which other etiologies and/or sources of liver injury are previously ruled out (Figure 1). In any case, each patient must be managed in a flexible, individualized way, especially in the presence of pre-existing chronic liver disease [21], as further described below.

Finally, the use of vaccines against COVID-19 has been associated with the appearance of two syndromes with hepatic manifestations: thrombotic thrombocytopenia and acute liver injury, similar to autoimmune hepatitis. Thrombotic thrombocytopenia has been associated with adenovirus-based vaccines and seems to be mediated by antibodies against platelet factor 4 [22, 23]. Liver injury similar to autoimmune hepatitis was first described in the context of messenger RNA vaccines, and later with the other type of vaccine [23], [24], [25]. It is a diagnosis of exclusion, and it is difficult to demonstrate a causative effect of vaccination on the development of autoimmune hepatitis, since vulnerable patients may develop it spontaneously concomitantly to immunization [23], [24], [25]. The two syndromes are rare complications, with an estimated frequency of 1 in 1 million of vaccinations, and do not affect general recommendations about vaccination [23].

## Pathogenesis of liver injury

SARS-CoV-2 spike protein (S) binds angiotensin converting enzyme 2 (ACE2) for virus entry into the target cells [15]. The S protein is activated by the transmembrane serine protease 2 (TMPRSS2) of the host cell for entry into the cell [26]. Notably, there is a significant association between the different variants of this protease and disease prognosis; therefore, their inhibition emerges as a potential therapeutic target [27].

Hepatic ACE2 expression has been documented in the epithelial cells of bile ducts, known as cholangiocytes (comparable to ACE2 expression in Type II pneumocytes, 60%); sinusoidal endothelial cells and hepatocytes (3%). Hepatic ACE2 expression can increase with liver injury and inflammation [28]. The high ACE2 expression in cholangiocytes, which is significantly higher than in hepatocytes (59.7% vs. 2.6%) [28], [29], [30], contrasts with the absence of a cholestatic biochemical profile, as mentioned above.

**Figure 2:**
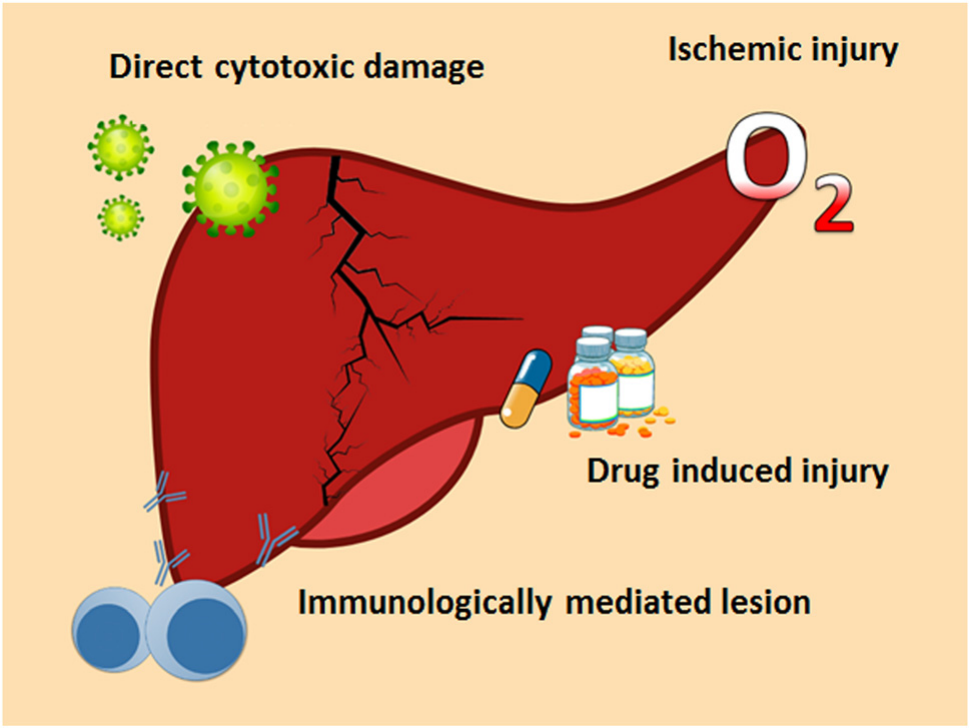
Mechanisms that induce liver damage in patients SARS-CoV-2 infection.

Liver injury due to COVID-19 is defined as any liver damage caused during the course of the disease and treatment [28]. In most cases, pathogenesis seems to be multifactorial (Figure 2), with several mechanisms being involved, such as direct cytotoxicity from replication of the virus in the liver, immune hyperactivation, ischemic damage, and drug-induced toxicity [29].

COVID-19-induced hepatocellular damage is characterized by the presence of moderate steatosis, lobular and portal inflammation, apoptotic/necrotic foci, and elevated levels of ALT and AST in plasma. This lesion could be caused by the direct replication of the virus in the liver, which induces mitochondrial dysfunction and stress in the endoplasmic reticulum, thereby contributing to steatosis, and ultimately causing liver damage [28].

SARS-CoV-2 encodes several structural and non-structural proteins (NSPS) that suppress antiviral response, which is primarily mediated by interferon through multiple mechanisms [31]. For instance, NSP1 inhibits translation in the host cell [32]. Additionally, SARS-CoV-2 infection could also activate mTOR, which ultimately inhibits autophagy (as a mechanism of viral degradation) and helps the virus escape from the immune system [28]. These viral proteins have not been demonstrated to exert a direct action on liver function. Anyway, this is not conceivable, given the ability of the virus to alter protein activity in the host cell [31, 33]. The direct cytopathic effect of the virus on ACE2-expressing cells and TMPRSS2 in the liver would explain most of direct liver damage. Therefore, the most affected cells in the liver are cholangiocytes, which have physiological functions in the adaptive immune response and regeneration of the liver [10, 34].

Other authors dismiss the idea that the virus exerts a direct action on the liver on the grounds that the association between liver biomarkers and prognosis is due to a pre-existing immune deregulation. This deregulation affects interaction of intrahepatic cytotoxic T-cells and Kupffer cells, as documented in other viral respiratory infections, and in the absence of intrahepatic replication [20].

The histopathological data currently available do not shed light on this issue. In some studies, the histopathological patterns analyzed, combined with the presence of viral RNA, seem to prove that liver injury is induced by the virus [35], [36], [37]. The most frequent post-mortem changes include steatosis (prevailingly macrovesicular), mild acute hepatitis (neuroinflammatory lobullar involvement), and mild portal inflammation [29, 36]. However, other authors support that the liver should not be a focus of concern, since the elevated levels of transaminases found in some patients seem to be related to endothelial dysfunction and coagulopathy [38, 39].

Although this evidence seems to be contradictory, it is not but a reflection of the multifactorial origin of liver injury induced by COVID-19. Therefore, its histopathological pattern is not specific of an etiology.

SARS-CoV-2 is associated with systemic inflammation, characterized by high levels of proinflammatory cytokines, which could explain the elevation of transaminases. Thus, levels of ALT seem to correlate with CRP, D-dimer, ferritinin or IL-6 [15]. Hyperinflammatory immune response, the so-called cytokine storm, may be more harmful than the cytopatic effect of the virus. In this sense, IL-6 correlates with the course of the disease; therefore, its inhibition (e.g. with tocilizumab) emerges as a therapeutic approach to this severe complication [6, 15]. In addition, immune dysfunction is crucial during the process of fibrosis [40], and inflammation induced by the virus could contribute to cases of fibrosis observed in COVID-19 patients [41].

Ischemic damage may play a role in liver injury. Thrombotic are frequent in the context of COVID-19 and are associated with poor prognosis. Hyperinflammation may favor immunologically-mediated thrombosis, primarily affecting microcirculation [38]. Intrahepatic microvascular thrombosis, which may cause hepatic hypoperfusion in COVID-19 patients, has a prevalence of 29%. Other contributing factors include hepatic congestion secondary to cardiomyopathy or systemic hypoxia, already observed in other viral pneumonias [6, 15].

Moreover, the medications used during the course of the disease may contribute to liver injury, which reported cases of injury associated with the use of remdesivir, lopinavir or tocilizumab [6, 15]. Notably, the patients at a higher risk of developing severe toxicity are patients with pre-existing chronic liver disease or liver transplant recipients, who may experience unintended drug-to-drug interactions with immunosuppressive agents [6, 15].

## Course of COVID-19 in patients with chronic liver disease

The prevalence of pre-existing chronic liver disease in large series of hospitalized patients with severe COVID-19 ranges between 0.6 and 1.4% [42], [43], [44]. Although COVID-19 prognosis is highly dependent on the nature of liver disease, it has been reported that around 60% of patients with chronic liver disease developed severe infections, with mortality rates reaching 18% [45].

The metabolic alterations found in patients with obesity or metabolic dysfunction-associated fatty liver disease (MAFLD), along with other risk factors such as age or male sex, have been associated with a poorer prognosis [46], [47], [48]. Additionally, abnormal liver biochemistry in patients with metabolic dysfunction are more significant during hospitalization [47, 49]. Nevertheless, it should be taken into account that these patients usually have comorbidities including hypertension, diabetes, cardiovascular disease or chronic obstructive pulmonary disease; therefore, the role of chronic liver disease in the development of severe COVID-19 should be analyzed with caution [50].

Hepatic cirrhosis is one of the factors associated with a poorer prognosis of COVID-19, which also occurs in other infections. As the grade of cirrhosis increases, based on Child-Pugh stages, mortality and ICU hospitalizations augment [9, 51]. Moreover, SARS-CoV-2 infection itself triggers hepatic decompensation, which favors the development of systemic inflammation, encephalopathy, deregulation of the immune system, and coagulopathy. The international registers SECURE-Cirrhosis and COVID-HEP provide updated data for large series of patients with cirrhosis and COVID-19 (https://www.covid-hep.net/updates.html). According to these registers, mortality in these patients reaches 38% (about 5 times higher than in the age-matched general population), rising to 70% in patients with Child-Pugh stage C [9]. Although the main cause of death was respiratory failure, acute hepatic decompensation occurred in almost half of the patients with cirrhosis, of whom 21% did not have respiratory symptoms [9].

Chronic liver diseases induced by hepatotropic viruses such as hepatitis B virus do not seem to be associated with a poorer prognosis of COVID-19 [52]. However, the immunosuppressive therapy used to fight the cytokine storm (glucocorticoids, tocilizumab, etc.) may increase the risk of reactivation of the virus [6]. These agents have been proven to induce a reversible elevation of transaminase levels [53] and their use is not recommended in the presence of AST and/or ALT levels five times above the ULN [54].

In the case of autoimmune hepatitis, this disease does not cause an increase in mortality with respect to the general population. The fact that this disease is treated with immunomodulatory or immunosuppressive agents does not involve a higher risk for complications associated with COVID-19. Therefore, it is not recommended that immunosuppressive treatment is reduced during the course of the disease [15].

The impact of hepatocellular carcinoma on COVID-19 prognosis has not been sufficiently documented. Immunosuppressive therapies and chemotherapeutic agents may favor SARS-CoV-2 infection and the development of severe disease, which suggests a poor prognosis [55].

With respect to liver transplant (LT) recipients, the pandemic has caused a substantial reduction in the number of liver transplants. This is due to the high morbidity and mortality of LT recipients who develop SARS-CoV-2 infection, added to the need to reserve ICU beds for patients with severe COVID-19, and the potential higher risk for SARS-CoV-2 infection associated with the initiation of immunosuppressive therapy [50]. The decrease in the number of donors, along with the risk for hospital-acquired SARS-CoV-2 infection, have limited transplants to severe cases at risk of developing life-threatening liver failure [6]. Additionally, LT recipients are primarily male, with a mean age of 56 years and a high prevalence of diabetes and obesity, which are associated with severe COVID-19. Further studies are necessary to understand the factors that make LT recipients more susceptible to SARS-CoV-2 infection, including a higher risk for re-infection due to a poor adaptive immune response, duration of neutralizing IgG, viral replication time, or the development of other rare symptoms [15].

## Management of COVID-19 in patients with chronic liver disease

There are some recommendations for the management of patients with chronic liver disease according to the etiology of the disease. In general, it is recommended to reduce social interactions as much as possible and use telemedicine for routine appointments [50].

MAFLD requires a more intense intervention in the lifestyle of patients, involving losing weight and improving diabetes control, to prevent metabolic imbalance, which may exacerbate COVID-19 disease. Despite being ACE inhibitors, therapies for hypertension must be maintained. In case of SARS-CoV-2 infection, early hospitalization is recommended [21].

Antiviral treatment should be maintained in patients with chronic hepatitis B or C, since it is not associated with a higher risk for SARS-CoV-2 infection. Prior to the use of potent immunosuppressive agents, it is recommended to determine the serologic status of the patient due to viral reactivation risk and consider the administration of a prophylactic antiviral therapy [6, 21].

Patients with hepatic cirrhosis are at a higher risk of developing severe COVID-19. Thus, in the presence of hepatic decompensation, testing for SARS-CoV-2 is necessary irrespectively of whether the patient exhibits respiratory symptoms or not. In case of infection, even with mild symptoms, hospitalization and immediate initiation of antiviral therapy is recommended [15, 56]. In addition, immunization against influenza and *Streptococcus pneumoniae* is recommended in these patients [21].

On the other hand, cirrhosis and the resultant immune dysfunction have been associated with poor response to other vaccines. Further studies are necessary to assess the efficacy of the different vaccines against SARS-CoV-2 and the potential need to adjust doses and/or intervals in this population [57].

It is necessary to follow up patients with hepatocarcinoma or at a higher risk of developing it (cirrhosis, chronic hepatitis, among others), since COVID-19 has a poorer prognosis in these patients. However, the incidence rate of hepatocarcinoma has not declined during the pandemic [15].

With regard to LT recipients, it is recommended to maintain immunosuppressive therapy. The dose should only be reduced to prevent lymphopenia or opportunistic infections in case of SARS-CoV-2 infection, without increasing the risk for graft rejection. Consider potential interactions between calcineurin inhibitors and the treatment for SARS-CoV-2 infection. Co-administration of monoclonal antibodies is not recommended, otherwise, concentrations of the immunosuppressive agent should be monitored [21, 29]. The generation of antibodies in response to immunization against SARS-CoV-2 is slower in LV recipients [15].

As to the use of vaccines in patients with chronic liver disease, hepatobiliary cancer or liver recipients, the EASL issued some recommendations that are regularly revised based on the latest evidence available. These recommendations include the immunization and prioritization of these patients and their caregivers. Likewise, prospective registers should be established for monitoring the safety, immunogenicity and effectiveness of the different vaccines in patients with chronic liver disease and liver transplant recipients [58].

## Conclusions

The effect of COVID-19 on the liver is unclear. There is cumulative evidence of a multifactorial cause of liver injury secondary to viral infection. Although liver alterations are frequent during the course of COVID-19, it is not well understood whether they are the cause or the effect of a poorer course of the infection. Otherwise, failure to evaluate the potential effects of COVID-19 on the liver, especially in patients with pre-existing liver disease or receiving immunosuppressive agents, may have fatal effects. Further studies are necessary to assess the prognostic value of abnormal liver biochemistry. Establishing a specific approach and/or treatment for liver alterations should be considered. Finally, patients with an underlying liver disease could benefit from studies evaluating their response to immunization, especially in a context where new variants of the virus have emerged. The establishment of international multicentric registers may facilitate this task, which should be complemented with molecular and translational studies.
